# Deficiency of ataxia‐telangiectasia mutated kinase attenuates Western‐type diet‐induced cardiac dysfunction in female mice

**DOI:** 10.14814/phy2.15434

**Published:** 2022-09-19

**Authors:** Mary C. Wingard, Suman Dalal, Paige L. Shook, Paulina Ramirez, Muhammad U. Raza, Patrick Johnson, Barbara A. Connelly, Douglas P. Thewke, Mahipal Singh, Krishna Singh

**Affiliations:** ^1^ Department of Biomedical Sciences James H Quillen College of Medicine, East Tennessee State University Johnson City Tennessee USA; ^2^ Department of Health Sciences East Tennessee State University Johnson City Tennessee USA; ^3^ Center of Excellence in Inflammation, Infectious Disease and Immunity Johnson City Tennessee USA; ^4^ Research and Development Service James H Quillen Veterans Affairs Medical Center Mountain Home Tennessee USA

**Keywords:** apoptosis, ATM, female, heart, Western‐type diet

## Abstract

Chronic consumption of Western‐type diet (WD) induces cardiac structural and functional abnormalities. Previously, we have shown that WD consumption in male ATM (ataxia‐telangiectasia mutated kinase) deficient mice associates with accelerated body weight (BW) gain, cardiac systolic dysfunction with increased preload, and exacerbation of hypertrophy, apoptosis, and inflammation. This study investigated the role of ATM deficiency in WD‐induced changes in functional and biochemical parameters of the heart in female mice. Six‐week‐old wild‐type (WT) and ATM heterozygous knockout (hKO) female mice were placed on WD or NC (normal chow) for 14 weeks. BW gain, fat accumulation, and cardiac functional and biochemical parameters were measured 14 weeks post‐WD. WD‐induced subcutaneous and total fat contents normalized to body weight were higher in WT‐WD versus hKO‐WD. Heart function measured using echocardiography revealed decreased percent fractional shortening and ejection fraction, and increased LV end systolic diameter and volume in WT‐WD versus WT‐NC. These functional parameters remained unchanged in hKO‐WD versus hKO‐NC. Myocardial fibrosis, myocyte hypertrophy, and apoptosis were higher in WT‐WD versus WT‐NC. However, apoptosis was significantly lower and hypertrophy was significantly higher in hKO‐WD versus WT‐WD. MMP‐9 and Bax expression, and Akt activation were higher in WT‐WD versus WT‐NC. PARP‐1 (full‐length) expression and mTOR activation were lower in WT‐WD versus hKO‐WD. Thus, ATM deficiency in female mice attenuates fat weight gain, preserves heart function, and associates with decreased cardiac cell apoptosis in response to WD.

## Graphical abstract

Deficiency of ataxia telangiectasia mutated kinase (ATM) in humans increases the risk of ischemic heart disease. Previously, we provided evidence that Western‐type diet in male ATM deficient mice accelerates body weight gain, induces systolic dysfunction with increased preload, and exacerbates cardiac hypertrophy, apoptosis, and inflammation. Here, we provide evidence that ATM deficiency associates with attenuated fat weight gain, and plays a cardioprotective role with preservation of heart function and decreased cardiac cell apoptosis in female mice fed with Western‐type diet. Further investigations of sex‐specific role of ATM in Western‐type diet‐induced cardiac functional and structural changes may help elucidate the appropriate sex‐specific treatment and nutritional counseling for patients with ATM deficiency.

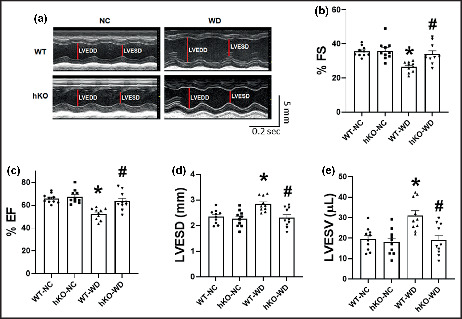

## 1 INTRODUCTION

Chronic consumption of a Western‐type diet (WD) has been shown to associate with changes in cardiac structural and mechanical function as well as low‐grade systemic inflammation, increased oxidative stress, and metabolic dysfunction (Kelsey et al., 2014; Liu & Lloyd, 2013; Lumeng & Saltiel, 2011). Obesity cardiomyopathy, which often occurs following extended consumption of WD and/or obesity, is characterized by functional and structural derangements, hemodynamic changes, and altered cardiac remodeling (Akki & Seymour, 2009; Alpert et al., 2014; Bhatheja et al., 2016; Gonçalves et al., 2016). The severity of obesity directly influences the change in blood volume, degree of hyperdynamic circulation, and associated preload and afterload dysfunction (Albakri, 2018a; Bhatheja et al., 2016). Therefore, deleterious changes in cardiac structure and function are often observed with obesity cardiomyopathy, which may include right and left ventricular eccentric/concentric hypertrophy, chamber dilation, increased fibrosis, systolic and diastolic dysfunction (Albakri, 2018a; Bhatheja et al., 2016). Further, studies show that animal models of obesity display diet‐induced cardiac dysfunction similar to humans such as biventricular stiffness, exacerbation of fibrosis, hypertrophy, plaque accumulation, and ischemic events (Abel et al., 2008; Bhatheja et al., 2016; Carbone et al., 2017; Gonçalves et al., 2016).

Ataxia telangiectasia mutated kinase (ATM) becomes activated in response to DNA double‐stranded breaks, oxidative damage, ionizing radiation, and other genotoxic mediators (Thrasher et al., 2017; Wingard et al., 2020). ATM is located in the nucleus, cytoplasm, and mitochondria, and therefore has a multitude of important functions (Espach et al., 2015; Thrasher et al., 2017; Wingard et al., 2020). Cell cycle regulation and DNA damage repair are important functions of ATM (Jia et al., 2017). However, ATM is actively involved in many other processes such as regulation of glucose metabolism, redox sensing, vesicle transport, autophagy, and peroxisome and mitochondrial function (Amirifar et al., 2019; Blignaut et al., 2019; Cheng et al., 2021; Halaby et al., 2008). Therefore, mutations that impair the function of ATM result in a multisystem disorder called ataxia‐telangiectasia (A‐T) (Rothblum‐Oviatt et al., 2016). While the severity of the A‐T phenotype is based on the type and frequency of mutation occurrence, neurological, endocrine and cardiovascular dysfunction are commonly observed (Nowak‐Wegrzyn et al., 2004; Rothblum‐Oviatt et al., 2016; Shiloh & Ziv, 2013; van Os et al., 2016). A‐T carriers, patients with mutations in one allele, account for approximately ~1.4%–2% of the population. A‐T patients exhibit an increased risk of metabolic dysfunction, cancer, and ischemic heart disease (Wingard et al., 2020).

While the cardiovascular effects of WD during ATM deficiency are not fully elucidated, we have previously provided evidence that WD during ATM deficiency in male mice associates with accelerated weight gain, systolic dysfunction with increased preload, exacerbation of cardiac remodeling including increased hypertrophy and apoptosis, and alterations in inflammatory and metabolic signaling (Wingard et al., 2021). The objective of this study was to investigate the role of ATM deficiency in WD‐induced changes in functional and biochemical parameters of the heart in female mice. The major finding of the study is that ATM deficiency in female mice attenuates fat weight gain, preserves cardiac function and associates with reduced cardiac cell apoptosis.

## 2 MATERIALS AND METHODS

### 2.1 Vertebrate animals and diets

This investigation follows the *Guide for the Care and Use of Laboratory Animals* published by the US National Institutes of Health (NIH Publication No. 85‐23, revised 1996). All experiments were executed following the protocols which were approved by the East Tennessee State University Committee on Animal Care. ATM deficient mice (129S6/SvEvTac) were acquired from Jackson Laboratory for breeding (stock #002753). ATM heterozygous knockout (hKO) mice were used for breeding as ATM KO mice have a limited lifespan (~2 months). All mice were genotyped prior to diet assignment and following exsanguination by PCR using primers suggested by the Jackson Laboratory. Age matched (~6 weeks old) WT and hKO female mice were placed on a normal chow (NC; Envigo 8604) or Western‐type diet (WD; Envigo TD 88137) for a 14‐week duration. The NC energy composition is—32% kcal protein, 14% kcal fat, 54% kcal carbohydrate and 4% sugar (by weight). The WD energy composition is—15.2% kcal protein, 42.0% kcal fat, 42.7% kcal carbohydrate and 34% sucrose (by weight). All mice had food and water available ad libitum, except when fasted for glucose level assessment, and were kept on a 12‐h‐dark/light cycle. Experiments were performed in blinded fashion whenever it was practically feasible.

### 2.2 Fasting glucose levels

When all groups reached the 14th week of NC or WD feeding, age‐matched mice from both genotypes were fasted for 4 h. A 5 mm Goldenrod lancet was used to gently nick the tail, which allowed collection of approximately 1 μl of blood sample. Blood glucose was then measured using a ReliOn monitoring system.

### 2.3 Echocardiography

Following the 14‐week duration of WD or NC, mice were anesthetized using a mixture of isoflurane (2%) and oxygen (0.6 L/min). After which, Vevo 1100 imaging system (VisualSonics, Fujifilm) equipped with a 22‐ to 55‐MHz MS550D transducer was used in order to assess the structural and functional parameters of the heart (Wingard et al., 2021). Transthoracic short axis view at mid‐papillary level view was used to obtain M‐mode recordings, while doppler tracings were acquired from the apical four chamber view. These recordings were used to measure and/or calculate the structural and functional parameters of the heart as described (Wingard et al., 2021).

### 2.4 Morphometric analysis

At the end of the study period, mice were weighed and anesthetized using a mixture of isoflurane (2%) and oxygen (0.6 L/min). The isolated hearts were perfused with Krebs–Henseleit buffer and arrested in diastole using KCL (30 mM). The hearts were then blotted to remove excess fluid and weighed. Collection and weighing of adipose tissue (subcutaneous and visceral fat) was performed via the removal of the epidermal skin layer post‐mortem. Hearts were divided into two transverse sections (base/mid and apex) and paraffin embedded. Mid‐cardiac transverse sections (5 μm thick) were used for the measurement of fibrosis using Masson's trichrome staining. Fibrosis was analyzed from 10 separate septal images using Nikon NIS software as described (Daniel et al., 2016). Percent fibrosis was calculated by division of the total fibrosis by the total area of each image multiplied by 100. To examine WD‐induced lipid accumulation, mid‐cardiac cryosections (10 μm thick) were stained with Oil red O, and analyzed as described (Wingard et al., 2021).

### 2.5 Western blot analysis

Heart lysates were prepared in RIPA buffer supplemented with Halt protease inhibitor cocktail as described (Wingard et al., 2021). Equal amounts of proteins (50 μg) were resolved by SDS‐PAGE and transferred to PVDF membranes. The membranes were blocked with 5% nonfat dry milk for 1 hour and incubated overnight with primary antibodies against MMP‐9 (1:1000, Cat# AB19016; Millipore), MMP‐2 (1:500, Cat# MAB3308; Millipore), Bax (1:1000, Cat# SC7480; Santa Cruz), PARP‐1 (1:1000, Cat# 9542; Cell Signaling), p‐Akt (ser473; 1:1000, Cat# 9271S; Cell Signaling), and p‐mTOR (ser‐2448; 1:1000, Cat# 5536S; Cell Signaling). The immune complexes were detected using appropriate secondary antibodies and chemiluminescent reagents (Wingard et al., 2021). Because assessment of loading using a total protein stain is considered as a better approach (Brooks & Lindsey, 2018); western blot data were normalized using Pierce reversible protein stain kit (Thermo Fisher). The portion of the membrane used for normalization is shown in Figures 6, 7, 8.

### 2.6 Terminal deoxynucleotidyl transferase nick end labeling (TUNEL) assay

Mid‐cardiac sections (5 μm thick) were stained using TUNEL kit as per manufacturer's instructions (In Situ Cell Death Detection Kit; Roche) followed by counterstaining with rhodamine‐conjugated wheat germ agglutinin (WGA, Rl‐1022; Vector) to visualize myocytes and Hoechst 33258 (10 μM; Sigma) to visualize nuclei (Daniel et al., 2014). Quantification of Hoechst‐positive nuclei was used as an index of total number of nuclei. TUNEL‐ and Hoechst‐positive staining observed within WGA‐stained cells allowed identification of myocytes. Ten images taken from the septal area/animal were used to measure the apoptotic index. Myocyte or total cell apoptosis was calculated as the percentage myocyte apoptotic nuclei or total cell apoptotic nuclei/total number of nuclei × 100 as described (Wingard et al., 2021).

### 2.7 Myocyte cross‐sectional area

WGA‐stained mid‐cardiac cross‐sections (5 μm thick) were also used to measure myocyte cross‐sectional area. For this, images were obtained with the EVOS M7000 imaging system and myocyte cross‐sectional area was quantified with Nikon NIS software as previously described (Wingard et al., 2021). Cross‐sectional area was measured using 10 different images from the septal area. Suitability of myocytes for cross‐sectional area was defined by circular cell body with a centrally located nucleus.

### 2.8 Cholesterol and triglyceride assay

Non‐hemolyzed serum was used for the measurement of total cholesterol and triglyceride levels according to manufacturer's instructions (Pointe Scientific). Sample to reagent ratio was diluted to 1:1000. Validity of the reaction was verified using serum standards provided by the manufacturer. Samples were read at a wavelength of 500 nm using a BioTek PowerWave XS2 microplate spectrophotometer. Levels of cholesterol and triglyceride were determined as: absorbance (sample)/absorbance (standard) multiplied by the concentration of standard.

### 2.9 Statistical analysis

All the data shown are expressed as means ± SE. Data were analyzed using two‐way analysis of variance (ANOVA) followed by Newman–Keuls test (GraphPad Prism 9 software) or 2‐tailed Student's *t*‐test. Probability (*p*) values of <0.05 were considered to be significant. The number (*n*) represents biological replicates.

## 3 RESULTS

### 3.1 Morphometric analyses

**FIGURE 1:**
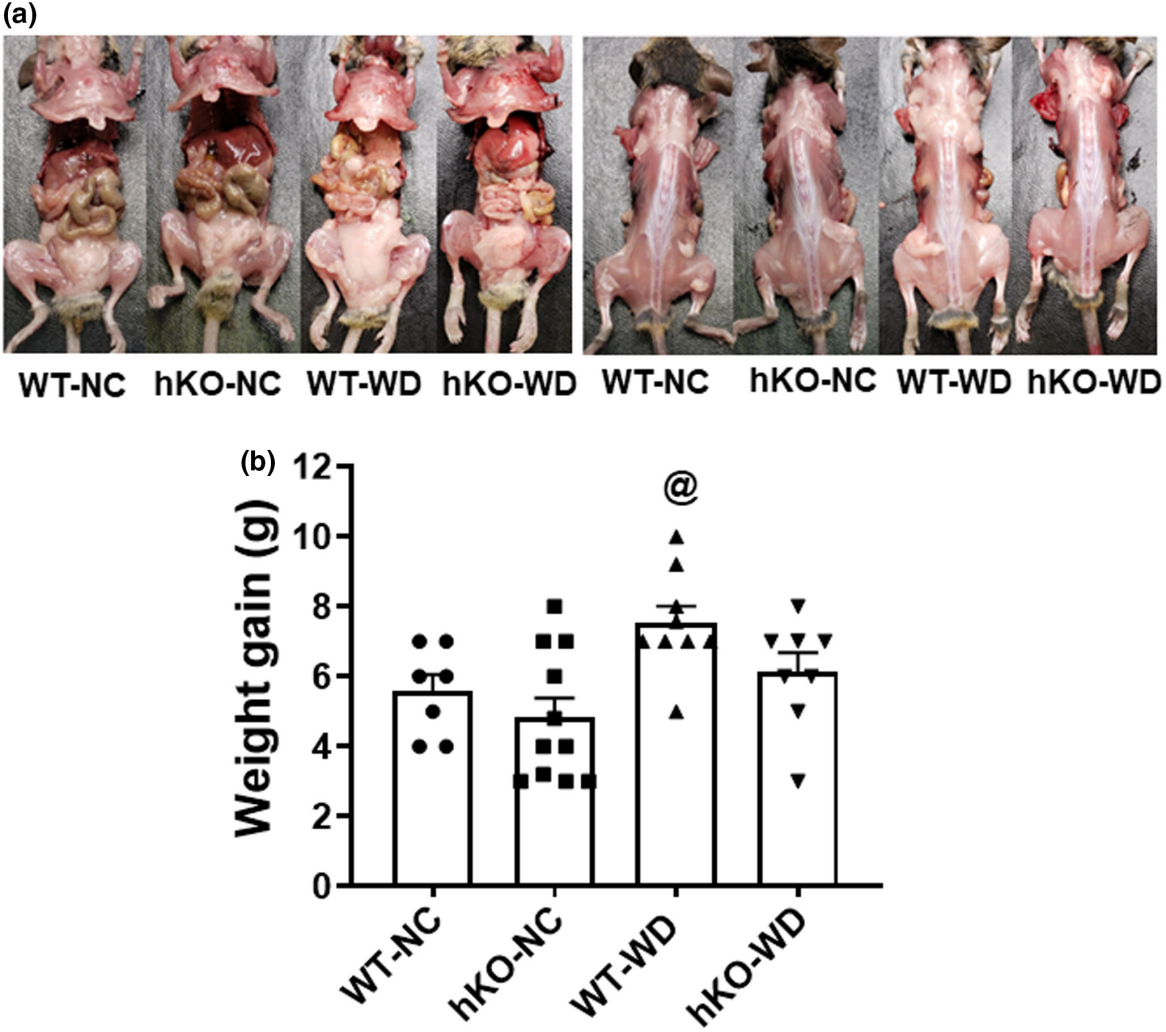
WD‐induced weight gain with time. (a) Visceral (left panel) and subcutaneous (right panel) adipose distribution after 14 weeks in NC or WD groups. (b) Weight gain for normal chow (NC) and Western‐type diet (WD) groups from week 0 to week 14. ^@^ *p* < 0.05 versus WT‐NC, *n* = 7–11; statistical analysis was performed using two‐tailed Student's *t*‐test.

Weight gain with concomitant increases in visceral and subcutaneous adipose tissue is commonly observed with WD (Gonçalves et al., 2016; Marwitz et al., 2015). Starting body weights (BW) were not different between the two genotypes (WT, 17.5 ± 0.4 g, *n* = 16; hKO, 18.1 ± 0.4 g, *n* = 19; *p* = NS). Feeding NC or WD for 14 weeks significantly increased BW in all four groups versus week 0 (WT‐NC, 23.6 ± 0.6 g, *n* = 7; hKO‐NC, 23.2 ± 0.6 g, *n* = 11; WT‐WD, 24.6 ± 0.6 g, *n* = 9; hKO‐WD, 23.9 ± 0.5 g; *n* = 8; Table 1) with no significant differences among the four groups. Using two‐tailed Student's *t*‐test, WD‐induced BW gain was significantly higher in WT group (Figure 1), not in hKO group. BW gain tended to be lower in hKO‐WD versus WT‐WD (WT‐WD, 7.5 ± 0.5 g, *n* = 9; hKO‐WD, 6.1 ± 0.5, *n* = 8; *p* = 0.07; Figure 1). WD significantly increased abdominal and total fat contents (normalized to BW) in both groups. Increase in subcutaneous fat was only observed in WT‐WD group. Interestingly, increase in subcutaneous, and total fat contents normalized to BW was significantly higher in WT‐WD versus hKO‐WD (Table 1). Heart weight (HW) and HW to BW (HW/BW) ratio remained unchanged among the four groups. WD led to a significant increase in serum cholesterol levels in both genotypes. However, serum cholesterol levels were significantly higher in hKO‐WD versus WT‐WD. Serum triglyceride levels were only increased in hKO‐WD versus hKO‐NC, and were significantly higher in hKO‐WD versus WT‐WD (Table 1). Oil red‐O‐staining displayed no visible lipid accumulation in the myocardium of WD groups (data not shown). Fasting glucose levels were higher in hKO‐WD versus WT‐WD (Table 1).

**TABLE 1:** Morphometric and biochemical measurements

Parameters	WT‐NC	hKO‐NC	WT‐WD	hKO‐WD
Body weight (g)	23.6 ± 0.6 (7)	23.2 ± 0.6 (11)	24.6 ± 0.6 (9)	23.9 ± 0.5 (8)
Heart weight (mg)	131 ± 5 (4)	133 ± 5 (5)	140 ± 7 (6)	136 ± 3 (3)
HW/BW ratio	5.6 ± 0.08 (4)	5.7 ± 0.3 (5)	5.7 ± 0.3 (6)	5.7 ± 0.1 (3)
SUBQ fat/BW ratio	16 ± 4.2 (4)	11 ± 2.3 (5)	38.2 ± 4.3 (5)*	17.3 ± 1.9 (4)^#^
ABD fat/BW ratio	17 ± 1.6 (4)	18.6 ± 2.9 (5)	43 ± 3.1 (5)*	33.9 ± 1.4 (4)^$^
Total fat/BW ratio	33 ± 4.1 (4)	29.6 ± 4 (5)	81.2 ± 6.1 (5)*	51.3 ± 0.9 (4)^$^ ^,^ ^#^
Total cholesterol (mg/dl)	80 ± 2.6 (6)	91 ± 6.4 (6)	164 ± 7.3 (6)*	214 ± 19.1 (6)^$^ ^,^ ^#^
Triglycerides (mg/dl)	40 ± 5.9 (6)	36 ± 7 (6)	59 ± 16 (6)	127 ± 8.6 (6)^$^ ^,^ ^#^
4‐hr Fasting Glucose (mg/dl)	133 ± 3.2 (10)	146 ± 1.6 (8)	130 ± 6.0 (10)	148 ± 6.4 (10)^#^

*Note*: Values are means ± SE. The number in parenthesis represents the number of animals used for each parameter. Data were analyzed using two‐way ANOVA followed by Newman–Keuls test.

Abbreviations: ABD, abdominal; BW, body weight; hKO‐NC, heterozygous knockout on normal chow; hKO‐WD, heterozygous knockout on western diet^$^; HW, heart weight; SUBQ, subcutaneous; WT‐NC, wild‐type on normal chow; WT‐WD, wild‐type on western‐type diet.

* *p* < 0.05 versus WT‐NC.

^$^ *p* < 0.05 versus hKO‐NC.

^#^ *p* < 0.05 versus WT‐WD.

### 3.2 Echocardiographic measurements

**FIGURE 2:**
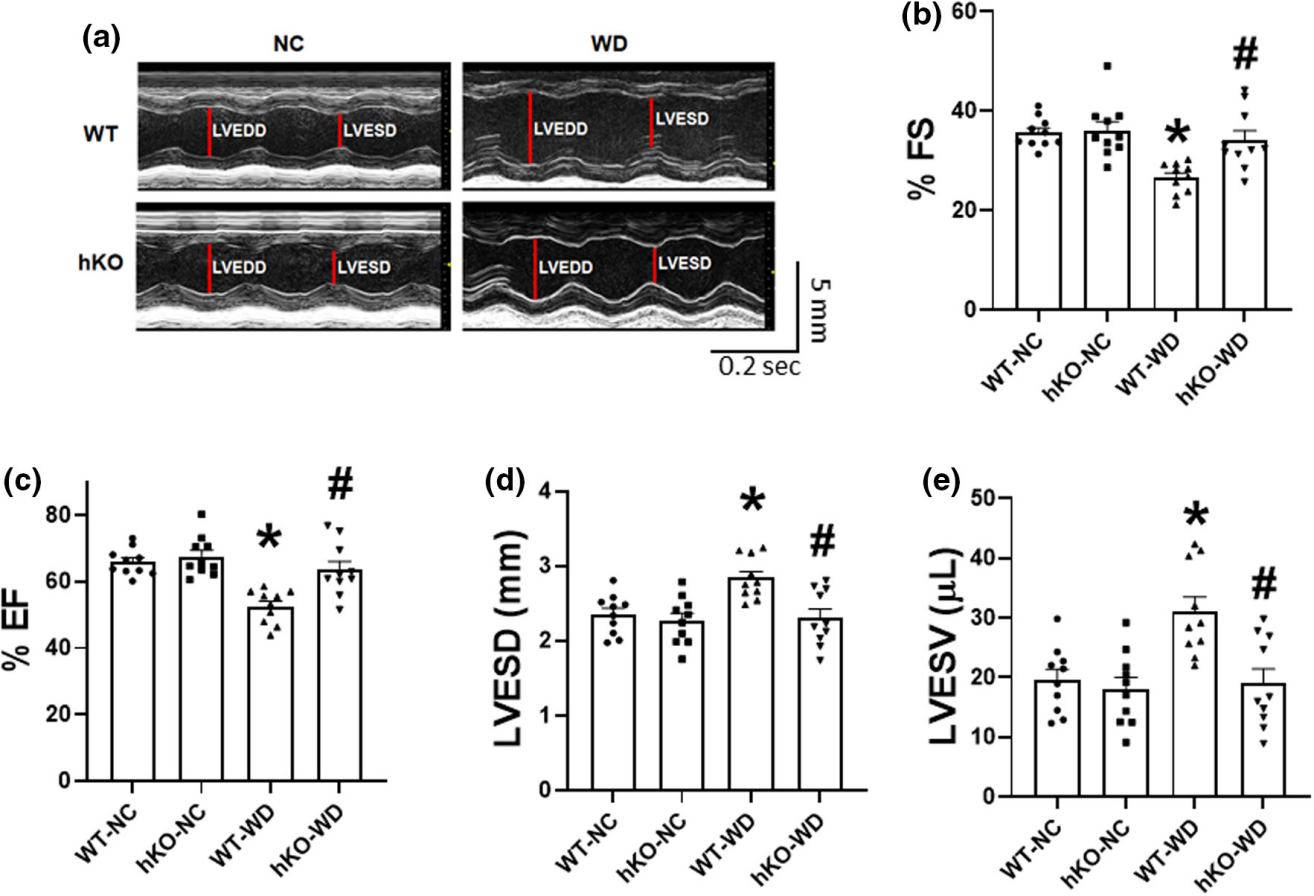
WD‐induced changes in M‐mode parameters of the heart. Indices of M‐mode parameters: % fractional shortening (%FS), % ejection fraction (%EF), LV end systolic diameter (LVESD), and LV end systolic volume (LVESV) were measured/calculated using M‐mode echocardiographic images following 14 weeks of NC or WD. (a) Representative M‐mode tracings for each group; (b) %FS; (c) %EF; (d) LVESD; (e) LVESV. *p < 0.05 versus WT‐NC, ^#^ *p* < 0.05 versus WT‐WD, *n* = 10. Statistical analysis was performed using two‐way ANOVA followed by Newman–Keuls test.

In male mice, WD decreased percent fractional shortening (%FS), ejection fraction (%EF) and increased LV end systolic diameter (LVESD) and volume (LVESV) to a similar extent in both WT and hKO groups (Wingard et al., 2021). Here, M‐mode echocardiography revealed no functional differences between the two NC groups. Heart rates remained unchanged among the four groups (Table 2). However, WD decreased %FS, and %EF, and increased LVESD and LVESV in WT‐WD group versus WT‐NC (Figure 2). Interestingly, no change in the functional parameters was observed in hKO‐WD versus hKO‐NC. In fact, %FS and %EF were significantly higher, and LVESD, and LVESV were significantly lower in hKO‐WD versus WT‐WD (Figure 2). Pulsed‐wave Doppler analysis showed a trend toward decrease in E wave in WT‐WD versus WT‐NC (WT‐NC, 513.62 ± 48.08; hKO‐NC, 486.61 ± 28.49; WT‐WD, 421.91 ± 36.65*; hKO‐WD, 497.51 ± 28.53; *p = 0.087 vs. WT‐NC; 2‐tailed Student's *t*‐test; *n* = 10–11; Table 2). Other parameters (A‐wave, AET, IVCT, IVRT and Tei Index) remained unchanged among the four groups (Table 2).

**TABLE 2:** PW echocardiographic parameters

Parameters	WT‐NC	hKO‐NC	WT‐WD	hKO‐WD
Heart rate (BMP)	352.9 ± 8.5	339.9 ± 10.3	334.1 ± 10.0	355.7 ± 7.8
E‐wave (mm/s)	531.62 ± 48.08	486.61 ± 28.49	421.91 ± 36.65	497.51 ± 28.53
A‐wave (mm/s)	344.60 ± 48.39	258.95 ± 15.59	255.18 ± 19.52	301.96 ± 23.29
E/A wave ratio	1.64 ± 0.09	1.89 ± 0.07	1.66 ± 0.08	1.76 ± 0.18
AET (ms)	59.07 ± 1.43	62.65 ± 1.52	58.77 ± 1.73	59.37 ± 1.93
IVCT (ms)	17.78 ± 1.10	19.13 ± 0.96	17.88 ± 0.86	17.98 ± 1.17
IVRT (ms)	23.45 ± 0.71	22.75 ± 0.65	24.40 ± 1.06	24.51 ± 1.06
Tei Index	0.69 ± 0.02	0.67 ± 0.02	0.73 ± 0.04	0.72 ± 0.04

*Note*: Values are means ± SE, *n* = 10–11. Data were analyzed using two‐way ANOVA followed by Newman–Keuls test.

Abbreviations: AET, aortic ejection time; IVCT, isovolumetric contraction time; IVRT, isovolumetric relaxation time.

### 3.3 Fibrosis, hypertrophy, and apoptosis

**FIGURE 3:**
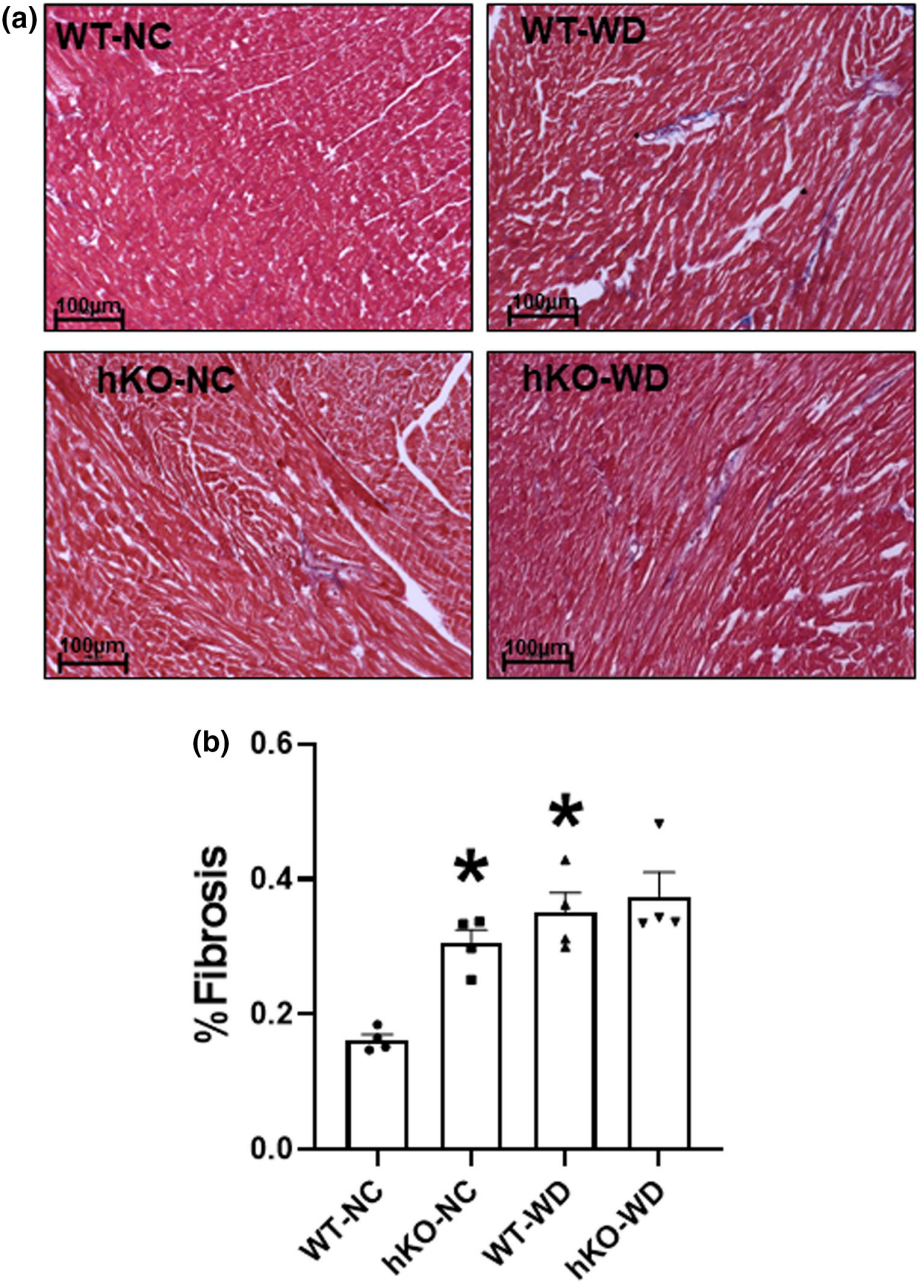
WD exacerbates fibrosis in wild‐type mice. (a) Masson's trichrome‐stained sections of the heart for each group and associated diet. Blue staining indicates fibrosis, while red staining indicates muscle tissue. (b) Quantitative measurements of fibrosis. *p < 0.05 versus WT‐NC, *n* = 4. Statistical analysis was performed using two‐way ANOVA followed by Newman–Keuls test.

WD is suggested to induce a variety of pathological modifications, which include increase in cardiac fibrosis, hypertrophy, and apoptosis (Akki & Seymour, 2009; Gonçalves et al., 2016; Kopp, 2019; Myles, 2014). WD and excessive adipose accumulation independently contribute to the development of cardiac hypertrophy (Albakri, 2018b; Gonçalves et al., 2016). Quantitative analysis of myocardial fibrosis using Massons trichrome staining showed a significant increase in percent fibrosis in hKO‐NC versus WT‐NC. WD led to a significant increase fibrosis in WT group, while no further increase in fibrosis was observed in hKO‐WD (Figure 3).

**FIGURE 4:**
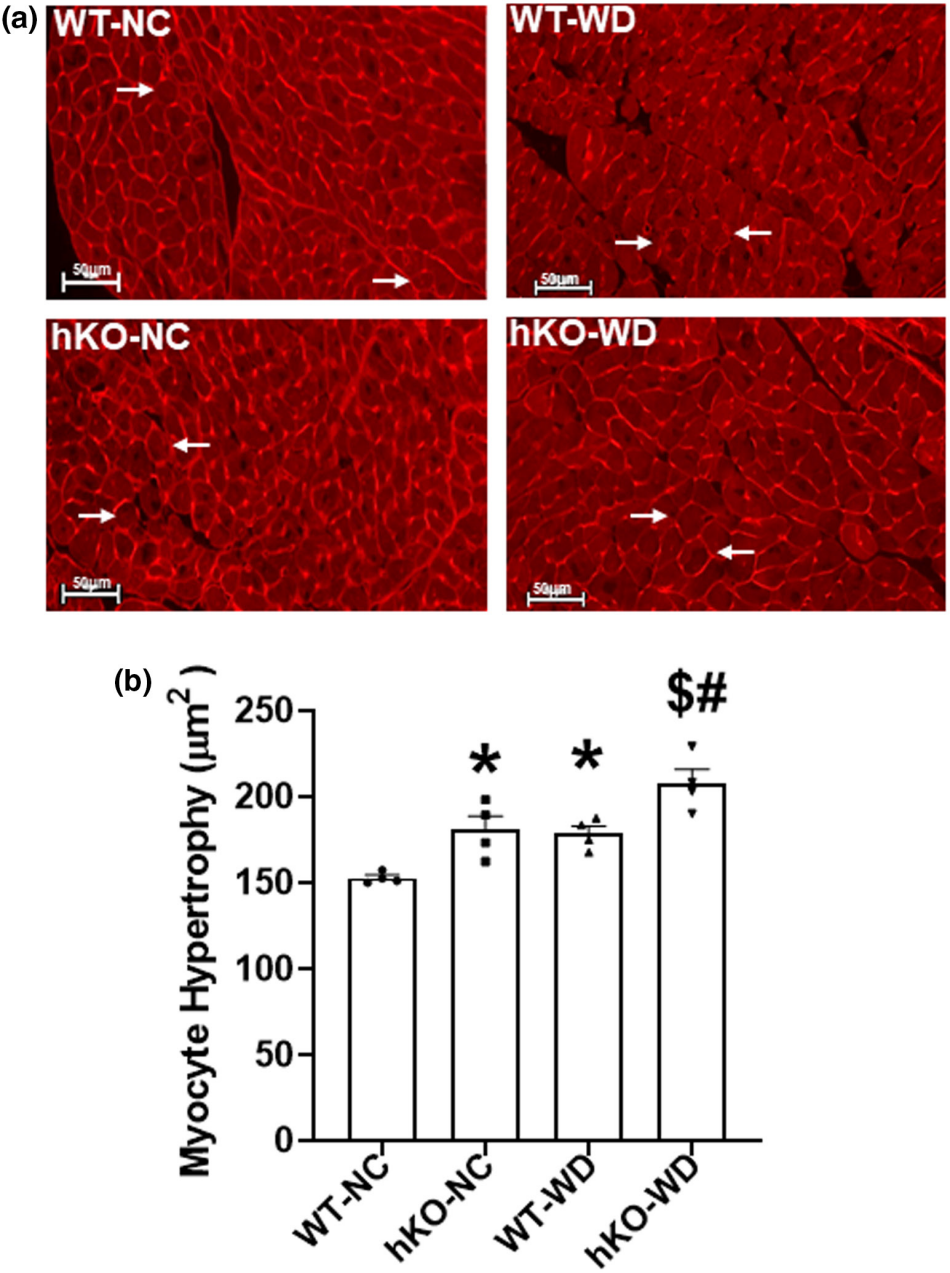
ATM deficiency increases myocyte hypertrophy in response to WD. (a) Representative images of wheat germ agglutinin (WGA)‐stained cross‐sections of the heart‐depicting myocytes. (b) Quantitative analysis of myocyte cross‐sectional area. *p < 0.05 versus WT‐NC, ^$^ *p* < 0.05 versus hKO‐NC, ^#^ *p* < 0.05 versus WT‐WD, *n* = 4. Statistical analysis was performed using two‐way ANOVA followed by Newman–Keuls test.

Hypertrophy, as assessed by the measurement of myocyte cross‐sectional area, was higher in hKO‐NC versus WT‐NC. WD significantly increased myocyte cross‐sectional area in both groups versus NC. However, myocyte cross‐sectional area remained significantly higher in hKO‐WD versus WT‐WD (Figure 4).

**FIGURE 5:**
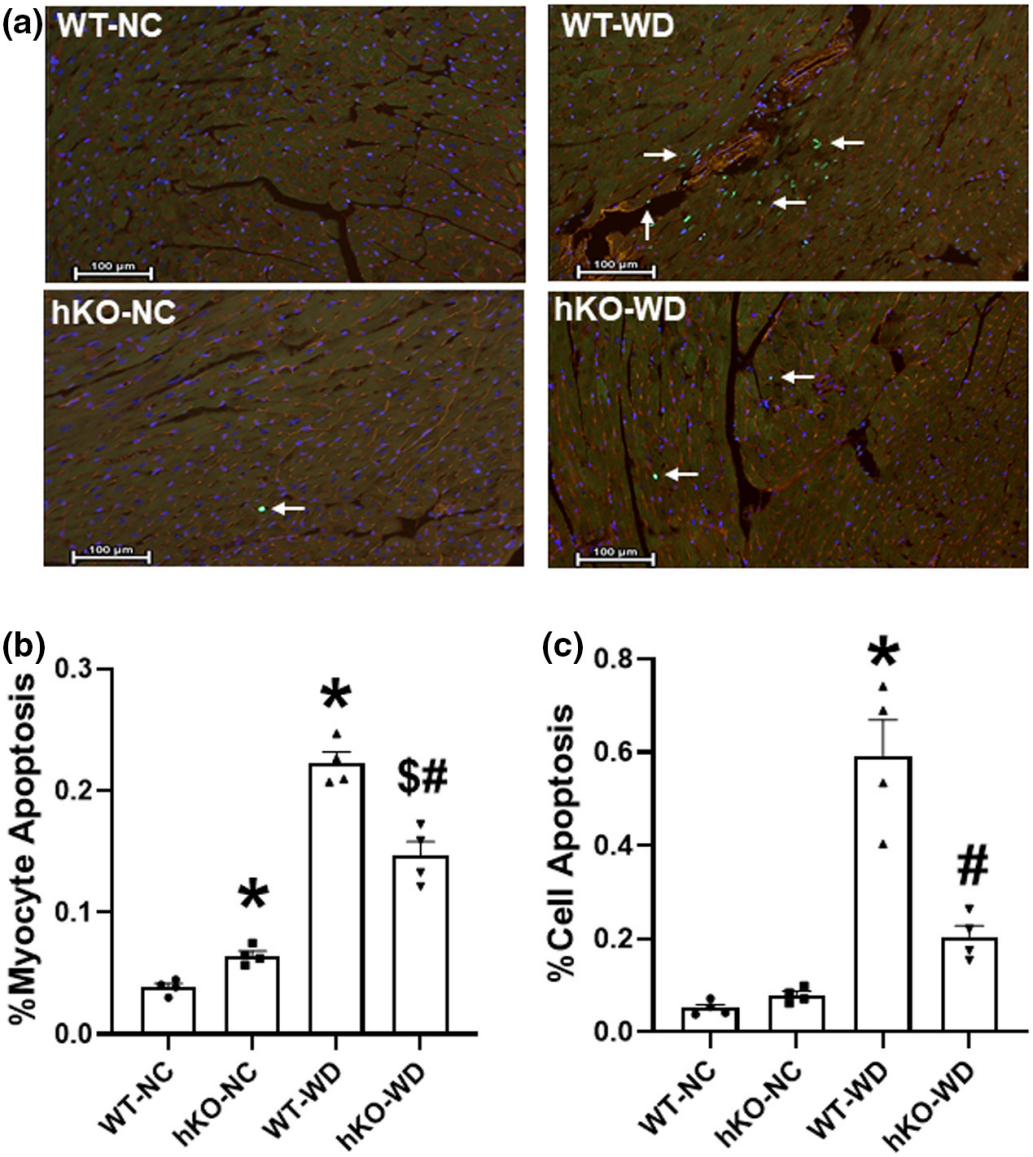
ATM deficiency attenuates apoptosis in the heart in response to WD. (a) Representative images of TUNEL (green), WGA (red) and Hoechst (blue) stained hearts. (b) Quantitative analysis of myocyte apoptosis. (c) Quantitative analysis of cardiac cell apoptosis. *p < 0.05 versus WT‐NC, ^$^ *p* < 0.05 versus hKO‐NC, ^#^ *p* < 0.05 versus WT‐WD, *n* = 4. Statistical analysis was performed using two‐way ANOVA followed by Newman–Keuls test.

Analysis of apoptosis using TUNEL assay showed increased myocyte apoptosis in hKO‐NC versus WT‐NC. WD induced a significant increase in myocyte apoptosis in both genotypes. However, increase in myocyte apoptosis was significantly lower in hKO‐WD versus WT‐WD (Figure 5b). Total cell apoptosis was significantly higher in WT‐WD versus WT‐NC and hKO‐WD (Figure 5c).

### 3.4 Expression of MMPs

**FIGURE 6:**
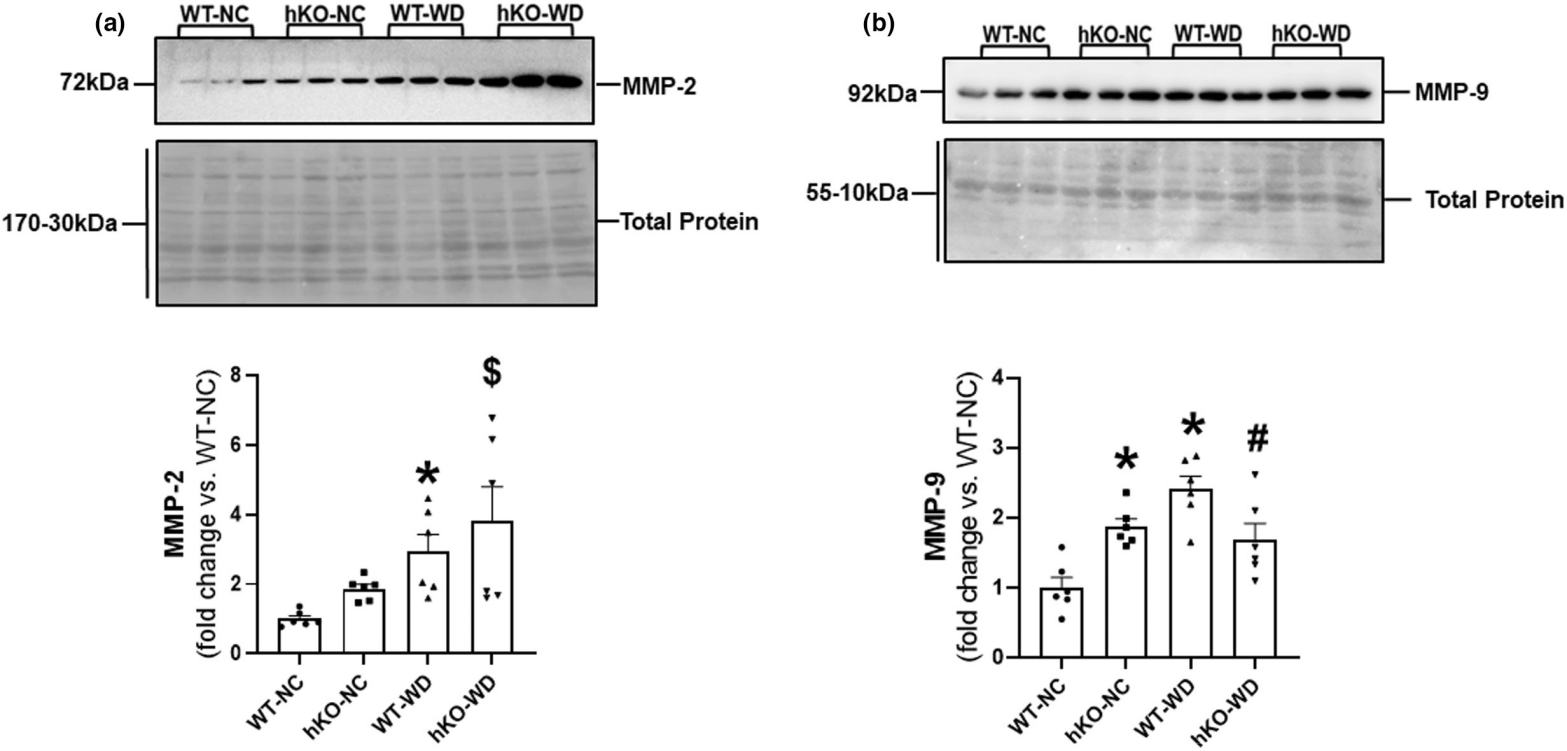
Expression of MMP‐2 and MMP‐9. Heart lysates were analyzed by western blots using anti‐MMP‐2 (a), and MMP‐9 (b) antibodies. Upper panels exhibit immunostaining for MMP‐2 and MMP‐9. Lower panels exhibit quantitative analyses normalized to total protein staining in each lane. *p < 0.05 versus WT‐NC, ^$^ *p* < 0.05 versus hKO‐NC, ^#^ *p* < 0.05 versus WT‐WD, *n* = 6. Statistical analysis was performed using two‐way ANOVA followed by Newman–Keuls test.

Cardiac fibrosis is commonly observed with WD and ATM deficiency (Daniel et al., 2014; Gonçalves et al., 2016; Liu et al., 2017). Excessive deposition of fibrosis can increase tensile scar strength and reduce myocardial contractile ability (Abel et al., 2008; Fredersdorf et al., 2004; Gonçalves et al., 2016; Martins et al., 2015; Sahraoui et al., 2016). Dysregulation of matrix metalloproteinase (MMP)‐2 and MMP‐9 has been observed with obesity, and contributes to the development of cardiovascular disease (Bäck et al., 2010; Jaoude & Koh, 2016; van Linthout et al., 2008). Western blot analysis of cardiac lysates using anti‐MMP‐2 antibodies showed no difference in MMP‐2 protein levels in hKO‐NC versus WT‐NC. WD significantly increased MMP‐2 expression in both genotypes with no difference between the two WD groups (Figure 6a). Protein levels of MMP‐9 were significantly higher in hKO‐NC versus WT‐NC. WD significantly increased protein levels of MMP‐9 only in WT‐WD versus WT‐NC. MMP‐9 expression was significantly lower in hKO‐WD versus WT‐WD (Figure 6b).

### 3.5 Expression and activation of proteins related to apoptosis and energy metabolism

**FIGURE 7:**
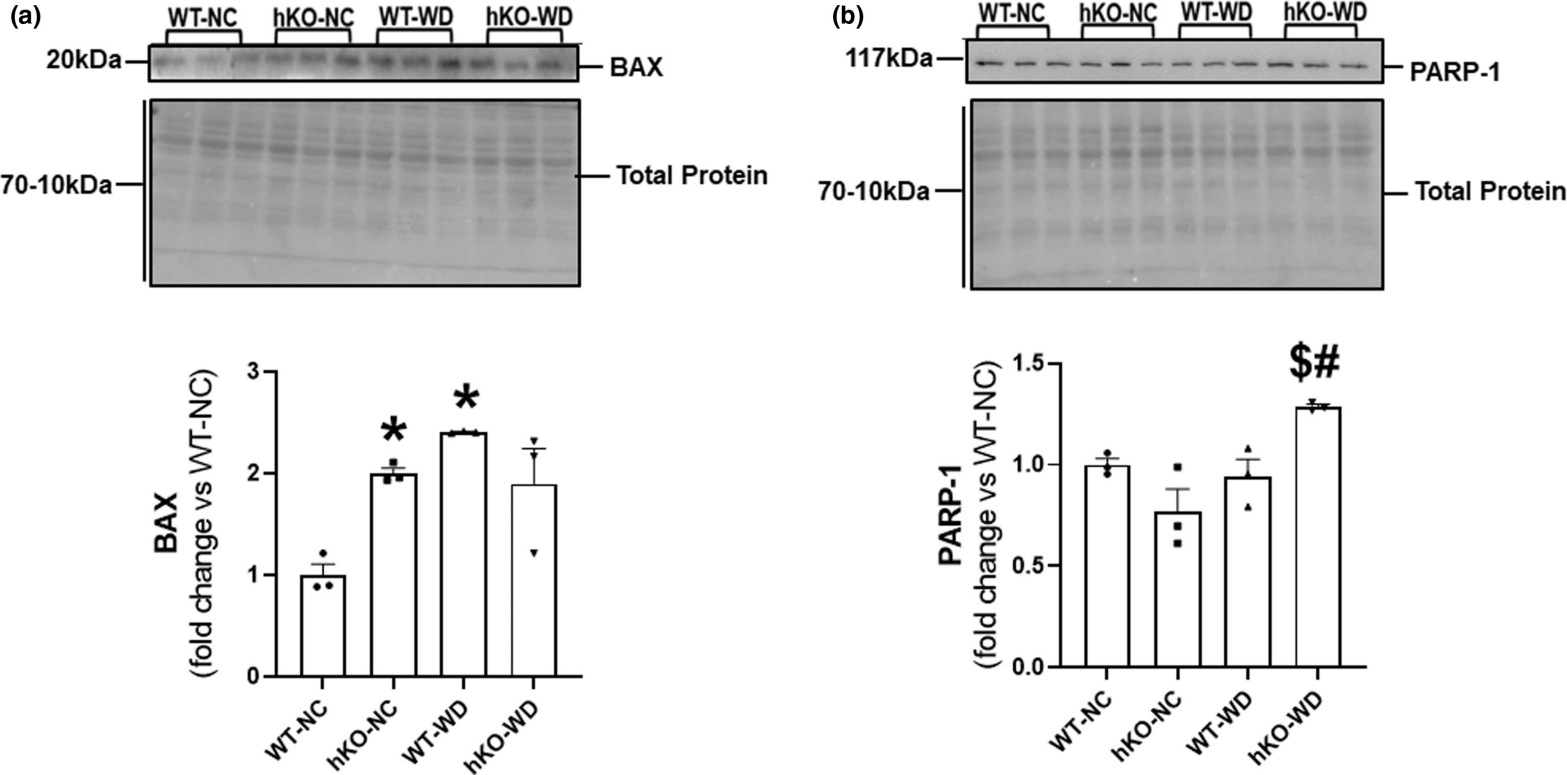
Expression of Bax and PARP‐1. Heart lysates were analyzed by western blots using anti‐Bax (a) and PARP‐1 (b) antibodies. Upper panels exhibit immunostaining for Bax and PARP‐1. Lower panels exhibit quantitative analyses normalized to total protein staining in each lane. *p < 0.05 versus WT‐NC, ^$^ *p* < 0.05 versus hKO‐NC, ^#^ *p* < 0.05 versus WT‐WD, *n* = 3. Statistical analysis was performed using two‐way ANOVA followed by Newman–Keuls test.

Diets high in fat associate with increased expression of Bax, a pro‐apoptotic protein, in the myocardium (Ballal et al., 2010). Western blot analysis of heart lysates showed that Bax expression is higher in hKO‐NC versus WT‐NC. WD significantly increased Bax expression in WT‐WD versus WT‐NC. However, no change in Bax expression was observed in hKO‐WD versus hKO‐NC (Figure 7a). PARP‐1 is an important DNA damage senor for single and double stranded DNA breaks (Pacher & Szabó, 2007). Western blot analysis showed that WD increases PARP‐1 (117 kDa) protein levels only in hKO group, and PARP‐1 protein levels were significantly higher in hKO‐WD and WT‐WD (Figure 7b).

**FIGURE 8:**
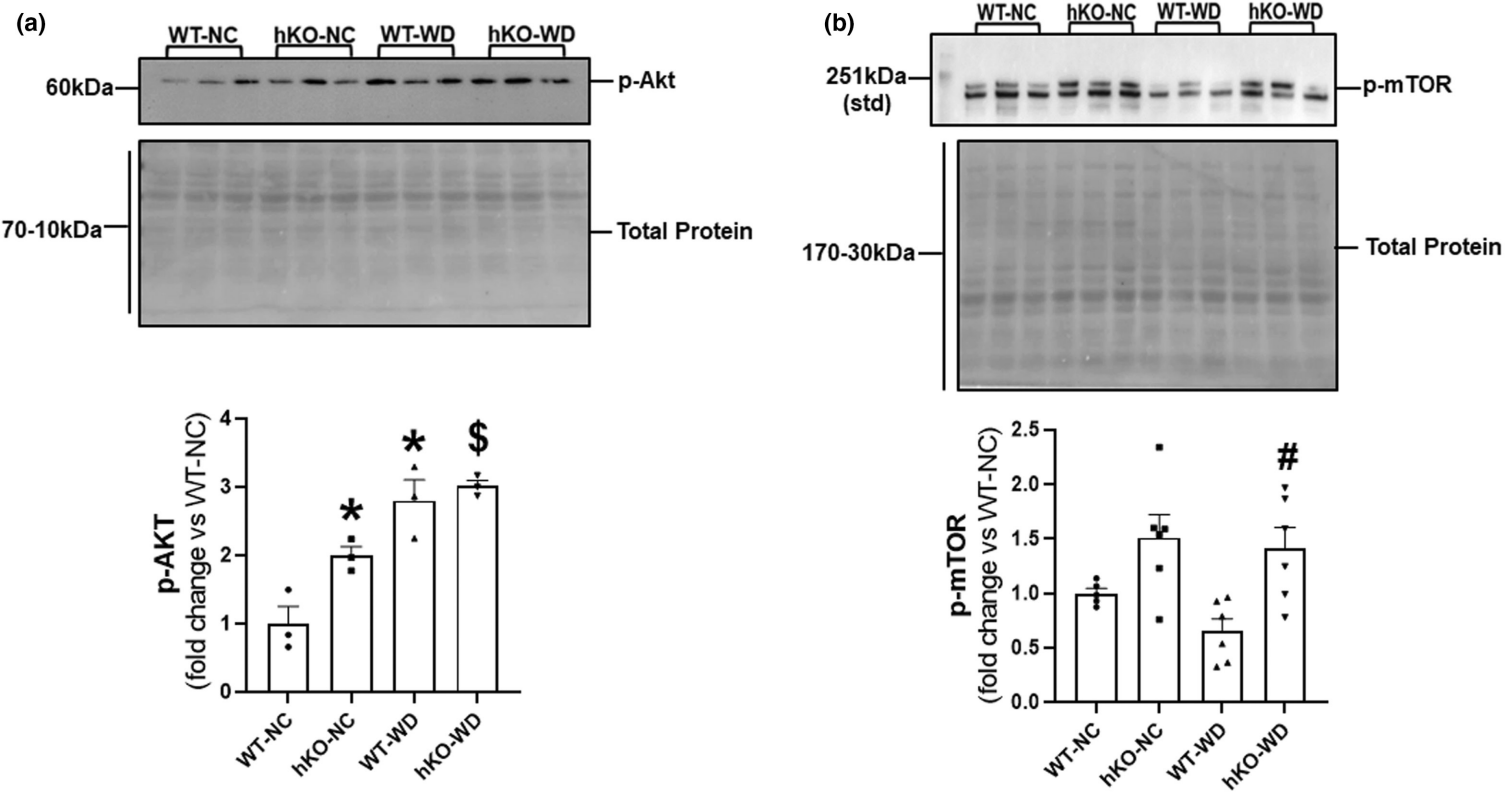
Activation of Akt and mTOR. Heart lysates were analyzed by western blots using anti‐p‐Akt (a) and p‐mTOR (b) antibodies. Bar graphs exhibit quantitative analyses normalized to total protein in each lane. *p < 0.05 versus WT‐NC, ^$^ *p* < 0.05 versus hKO‐NC, ^#^ *p* < 0.05 versus WT‐WD, *n* = 3 for p‐Akt; *n* = 6 for p‐mTOR. Statistical analysis was performed using two‐way ANOVA followed by Newman–Keuls test. The p‐mTOR antibody recognized two proteins on SDS‐PAGE. Predicted molecular weight of mTOR is ~289 kDa. However, it runs ~230 kDa on SDS‐PAGE. Therefore, the upper band below 251 kDa molecular weight marker (std) is used for quantification.

High fat diet is shown to associate with increased phosphorylation of Akt in the heart without affecting its expression (Fang et al., 2008). Obesity and WD also modulate mTOR signaling, which may exacerbate insulin resistance and other obesity‐related diseases (Jia et al., 2014; Mao & Zhang, 2018). Western blot analysis of heart lysates showed significantly higher Akt phosphorylation (activation) in hKO‐NC versus WT‐NC group. WD induced an increase in Akt activation in both groups with no significant difference between the two WD groups (Figure 8a). mTOR phosphorylation (activation) was not significantly different between hKO‐NC versus WT‐NC. mTOR activation was found to be significantly higher in hKO‐WD versus WT‐WD (Figure 8b).

## 4 DISCUSSION

The majority (~60%) of the American population consume a WD and are overweight or obese (Centers for Disease Control and Prevention USD of H and HS. National Diabetes Statistics Report, 2017; Hales et al., 2017; Office of Disease Prevention and Health Promotion, 2019; NIDDK. The National Institute of Diabetes and Digestive and Kidney Diseases, 2017). A‐T carriers, make up ~1.4%–2.0% of the general population, are more vulnerable to ischemic heart disease. Therefore, consumption of WD can further increase the risk of developing heart disease in A‐T carriers (Guleria & Chandna, 2016; Thrasher et al., 2017; Wingard et al., 2020). Previously, we have shown that WD during ATM deficiency in male mice associates with accelerated weight gain, systolic dysfunction with increased preload and exacerbation of cardiac remodeling (Wingard et al., 2021). This study explored WD‐induced functional and biochemical alterations of the heart using female ATM deficient mice. The major findings of the study are that ATM deficiency in female mice‐ (1) attenuates fat weight gain; (2) preserves cardiac function; (3) reduces WD‐induced cardiac cell apoptosis; and (4) alters the expression and activation of signaling molecules associated with fibrosis, apoptosis, and metabolism; (5) increases triglycerides and cholesterol levels.

Increase in visceral and subcutaneous adipose tissue is a common consequence of long term WD consumption (Gonçalves et al., 2016; Wang & Liao, 2012). Evidence has been provided that lack of ATM attenuates fat accumulation (Takagi et al., 2015). Not much is known regarding the effect of WD on body weight and fat accumulation in A‐T carriers. Previously, ATM deficiency in male mice associated with accelerated weight gain during weeks 5, 6, 7, 8, and 10 in response to WD. At the completion of WD (14 weeks post‐WD), male WD‐fed ATM deficient mice exhibited no differences in adipose accumulation (subcutaneous and visceral), and total serum cholesterol and triglyceride levels (Wingard et al., 2021). Interestingly, female ATM deficient mice exhibited no significant increase in BW gain in response to WD, and BW gain tended to be lower in hKO‐WD versus WT‐WD (*p* = 0.07 vs. WT‐WD; 2‐tailed Student's *t*‐test). In addition, female ATM deficient mice exhibited lower fat contents (subcutaneous, and total), and increased serum levels of cholesterol and triglycerides 14 weeks post‐WD. The attenuation of WD‐induced fat accumulation in female ATM deficient mice is consistent with the previous report where lack of ATM is shown to associate with lower subcutaneous adipose tissue and differentiation (Takagi et al., 2015). In ApoE−/− background, ATM deficiency in mice associated with increased plasma cholesterol levels versus their control groups (Wu et al., 2005). A‐T patients are also reported to have increased plasma cholesterol and triglyceride levels (Andrade et al., 2015), and exhibit a variety of sex‐specific endocrine abnormalities (Nissenkorn et al., 2016). Pubertal and adult A‐T females have lower basal levels of estradiol (Nissenkorn et al., 2016; Zadik et al., 1978). In general, serum cholesterol and triglyceride levels are known to correlate negatively with estrogen in postmenopausal women (Mešalić et al., 2008). Together, it appears that ATM deficiency affects fat accumulation and plasma lipid levels in a sex‐specific manner, and the effects of ATM deficiency on adipocyte development and maturation may be more pronounced in female mice. This notion is supported by the observation of increased serum cholesterol and triglyceride levels in ATM deficient female mice as they may have a reduced ability to store excess energy as fat.

High fat diets and ATM deficiency independently associate with the development of insulin resistance (Abel et al., 2008; Albakri, 2018a; Wingard et al., 2020). In male mice, fasting blood glucose levels were not significantly different between the two normal chow groups (WT‐NC vs. hKO‐NC). WD significantly increased fasting blood glucose levels in male WT group, not in hKO (Wingard et al., 2021). Here, we observed higher fasting blood glucose levels in ATM‐deficient female mice on WD versus WT‐WD. The observed increase in fasting blood glucose levels in ATM‐deficient female, not male, mice point toward the possibility of sex‐specific differences in insulin resistance with WD.

The severity and duration of excess fat accumulation and/or WD can directly influence the degree of cardiac mechanical and structural dysfunction (Abel et al., 2008; Bhatheja et al., 2016; Ebong et al., 2014; Gonçalves et al., 2016). The increased circulating blood volume observed in obesity can contribute to chamber dilation, leading to systolic dysfunction (Albakri, 2018a). Previously, we provided evidence that WD induces systolic dysfunction to a similar extent in WT and ATM‐deficient male mice as observed by a similar decrease in %FS and %EF and increase in LVESD and LVESV (Wingard et al., 2021). However, diastolic dysfunction was noted with increased afterload in WT‐WD male group, while hKO‐WD male group displayed increased preload. Here, WD‐induced systolic dysfunction was only observed in female WT mice as indicated by decreased %FS and %EF, and increased LVESD and LVESV. All these systolic parameters remained unchanged in ATM deficient group 14 weeks post‐WD. Increases in visceral adiposity is an independent predictor of the development of cardiovascular dysfunction (Abraham et al., 2015; Powell‐Wiley et al., 2021). Therefore, reduced WD‐induced fat accumulation in female ATM deficient mice may play an important role in the preservation of cardiac function. WD is shown to induce diastolic dysfunction in C57BL6 mice with increased IVRT (Bostick et al., 2015; Manrique et al., 2013). In these studies, diet was started when mice were 4 weeks old and WD constituted of 17.5% sucrose, 17.5% fructose, and 46% fat. In our study, WD feeding for 14 weeks tended to decrease peak E wave velocity in WT‐WD versus WT‐NC (*p* = 0.087 vs. WT‐NC; 2‐tailed Student's *t*‐test). However, other doppler parameters A wave, E/A wave ratio, AET, IVRT, IVRT, and Tei index were not found to be significantly different among the groups. It should be noted that our study used 129S6/SvEvTac mouse strain. WD feeding was started when mice were 6 weeks old, and WD contained 34% sucrose and 42% fat with no fructose. Therefore, it is possible that mouse strain, starting age of diet onset and composition of WD may affect functional parameters of the heart.

WD, adiposity and ATM deficiency independently associate with increased cardiac fibrosis, hypertrophy, and cardiac dysfunction (Albakri, 2018a; Daniel et al., 2014, 2016; Foster et al., 2011; Gonçalves et al., 2016). Previously, we demonstrated that ATM deficiency in male mice associates with increased fibrosis and hypertrophy at basal levels. WD induced a significant increase in fibrosis only in the male WT group. However, WD‐mediated increase in hypertrophy was greater in ATM‐deficient male mice versus WT (Wingard et al., 2021). Here, female ATM deficient mice also exhibit increased levels of fibrosis and hypertrophy at basal levels, findings consistent with the male counterparts. Also, WD significantly increased fibrosis and hypertrophy in WT‐WD versus WT‐NC. However, WD‐induced myocyte hypertrophy, as measured by increased myocyte cross‐sectional area, remained higher in ATM deficient female mice versus WT. Obesity with concomitant changes in hemodynamics can exacerbate fibrosis and hypertrophy (Martins et al., 2015). Further, the degree of adipose tissue accumulation correlates with increased cardiac remodeling (Albakri, 2018a; Gonçalves et al., 2016). As fibrosis generally precedes the development of hypertrophy (Albakri, 2018a), it is possible that the increased basal cardiac fibrosis and hypertrophy are serving as a protective mechanism against WD‐induced cardiac dysfunction in female ATM‐deficient mice.

MMP‐2 and MMP‐9 play an important role in fibrotic signaling and ECM degradation (Lindsey et al., 2016; Talman & Ruskoaho, 2016). MMP expression is tightly controlled to maintain balance between ECM degradation and synthesis (Talman & Ruskoaho, 2016). Consistent with our previous observation in male mice (Wingard et al., 2021), we observed increased basal expression of MMP‐9 in ATM‐deficient female hearts. WD led to a significant increase in MMP‐9 expression only in WT group. These studies provide evidence that both WD and ATM deficiency may play a role in modulation of cardiac fibrosis by affecting expression of fibrosis‐related proteins such as MMP‐9. The findings of increased MMP‐2 and MMP‐9 expression with increased fibrosis in WT‐WD appear counterintuitive. However, deposition of fibrosis is a complex and dynamic process involving synthesis, degradation, and deposition of extracellular matrix (ECM) proteins. In a pressure overload model of LV hypertrophy, increased MMP‐2 activity associated with increased fibrosis (Matsusaka et al., 2006). Targeted deletion of MMP‐9 decreases collagen deposition in the heart post‐MI (Ducharme et al., 2000). It should be noted that the observations of fibrosis and expression of MMPs were made 14 weeks post‐WD. Further investigations are needed to understand the role of ATM deficiency in deposition of myocardial fibrosis in response to WD. A time course analysis of fibrosis‐related proteins may help understand modulation of fibrosis in WT and ATM‐deficient heart post‐WD.

The continual increases in blood volume associated with obesity can induce excessive cardiac remodeling and lead to cardiac cell apoptosis (Albakri, 2018a). Further, WD and adiposity may exacerbate cardiac cell apoptosis due to lipotoxicity, decreased metabolic flexibility, and associated increase in toxic byproducts and increased oxidative stress (Akki & Seymour, 2009; Barouch et al., 2006; Carbone et al., 2015; Gonçalves et al., 2016; Kopp, 2019; Wende & Dale, 2010; Zeng et al., 2015). Previously, ATM deficiency in male mice associated with increased myocyte and total cell apoptosis at basal levels compared to WT. WD induced a significant increase in myocyte apoptosis in both genotypes. However, WD‐induced myocyte apoptosis was significantly higher (~2.5‐fold) in ATM deficient male mice versus their WT counterparts (Wingard et al., 2021). Here, ATM deficiency in female mice also associated with increase in myocyte apoptosis versus WT at basal levels. WD increased myocyte apoptosis in both genotypes. However, myocyte and total cell apoptosis were significantly lower in the myocardium of ATM‐deficient female mice versus WT. The lesser extent of apoptosis in ATM‐deficient female group may serve as an additional cardioprotective mechanism during WD‐induced cardiac remodeling.

Bax, a pro‐apoptotic protein, modulates mitochondrial membrane potential to initiate the apoptotic cascade (Daniel et al., 2014; Pawlowski & Kraft, 2000). In male mice, ATM deficiency associates with increased Bax expression in the myocardium under basal conditions. Interestingly, WD increased Bax expression in male WT‐WD, not hKO‐WD, group (Wingard et al., 2021). Similar to male mice, female ATM‐deficient mice also displayed increased Bax expression in the heart under basal conditions. WD increased Bax expression in WT‐WD versus WT‐NC group. Conversely, ATM deficient female group displayed no change in Bax expression in response to WD. Full length PARP‐1 (117 kDa) is a sensor of single‐ and double‐stranded DNA breaks, and is suggested to serve as regulator of DNA damage repair (Pacher & Szabó, 2007). Previously, we reported that WD decreases levels of intact PARP‐1 in ATM‐deficient male mice when compared to their WT counterparts (Wingard et al., 2021). Here, WD increased PARP‐1 protein levels only in ATM‐deficient group. Thus, increase in Bax expression with no increase in intact PARP‐1 protein levels suggest interference in DNA damage response in WT‐WD group, leading to increased cardiac cell apoptosis in WT‐WD group. On the other hand, increase in intact PARP‐1 protein levels may help explain the observed decrease in apoptosis in ATM‐deficient group in response to WD.

Diet induced obesity modulates important metabolism and cell survival proteins such as Akt and mTOR (Lyons & Roche, 2018; Mao & Zhang, 2018; Matsui et al., 2006). Interestingly, short‐term Akt activation can be cardioprotective as it promotes physiological hypertrophy. However, long‐term Akt activation associates with pathological hypertrophy with concomitant increase in fibrosis (Chaanine & Hajjar, 2011). Previously, ATM deficiency in male mice associated with increased activation of Akt at basal levels. Following WD, Akt activation increased in WT‐WD versus WT‐NC, while it remained unchanged in hKO‐WD versus hKO‐NC (Wingard et al., 2021). Here, Akt activation was also higher in ATM deficient female mice at basal levels. WD further increased Akt activation in both genotypes. The increased activation of Akt may help explain increased fibrosis and hypertrophy in these groups. Transgenic mice overexpressing mTOR are shown to display attenuated high fat diet‐induced fibrosis and cardiac dysfunction following ischemia–reperfusion injury (Aoyagi et al., 2015). Previously, ATM deficiency in male mice associated with increased mTOR activation at basal levels. Following WD, activation of mTOR was significantly lower in ATM‐deficient mice, and remained unchanged in WT group (Wingard et al., 2021). Here, mTOR activation was significantly higher in hKO‐WD versus WT‐WD. This higher mTOR activation observed in ATM deficient heart post‐WD may serve as an important step in the preservation of cardiac function.

In summary, the data presented here, in combination with our previous observations in male mice (Wingard et al., 2021), identify important sex‐specific differences during ATM deficiency, specifically in response to WD. The study provides first evidence that ATM deficiency in female mice attenuates WD‐induced adipose accumulation. In addition, ATM deficiency in female mice associates with preservation of cardiac function 14 weeks post‐WD. The preservation of heart function in ATM‐deficient females post‐WD may involve decreased myocyte apoptosis and increased myocardial hypertrophy. It should be noted, however, that all the observations were made 14 weeks post‐WD. Study duration, 14‐week post‐WD, was chosen to compare our previous findings of male mice (Wingard et al., 2021). It is possible that a different outcome with respect to functional and biochemical parameters may be obtained if study duration is extended beyond 14 weeks. Further, the effects of WD may vary with the age of diet onset (Salinero et al., 2018). In our study, the diet onset age of mice was 6 weeks. Future investigations are needed to examine the influence of other diet onset ages (young adult and middle‐aged) during ATM deficiency. It would also be interesting to investigate if endocrine system, specifically estrogen, plays a role in the preservation of cardiac function in ATM‐deficient WD‐fed female mice as postmenopausal women are more susceptible to developing obesity and metabolic complications versus premenopausal women (Curtis, 2009; Mauvais‐Jarvis et al., 2017). Use of ATM‐deficient mice suggest that ATM deficiency in female mice may contribute to lower body weight gain and improved heart function. However, future investigations are needed to dissect if prevention in body weight gain is responsible for cardioprotection or if reduced ATM deficiency directly affects heart function. Additionally, further understanding of underlying molecular mechanisms leading to preservation of heart function during ATM deficiency in WD‐fed female mice may help reveal important insights into the appropriate sex‐specific treatment and nutritional counseling of patients with ATM deficiency.

## AUTHOR CONTRIBUTIONS

M.C.W., M.S., and K.S. conceived and designed research; M.C.W., S.D., P.L.S., P.R., M.U.R., P.J., B.A.C., and D.P.T. performed experiments; M.C.W., P.L.S., and M.U.R, analyzed data; M.C.W., P.L.S., and K.S. interpreted results of experiments; M.C.W., P.L.S., and M.U.R prepared figures; M.C.W. drafted manuscript; M.C.W., S.D., P.L.S., M.S., and K.S. edited and revised manuscript; M.C.W., S.D., P.L.S., P.R., M.U.R., P.J., B.A.C., D.P.T., M.S., and K.S. approved final version of manuscript.

## FUNDING INFORMATION

This work was supported by Merit Review awards (I01BX004045 and I01BX002332) from the Biomedical Laboratory Research and Development Service of the Veterans Affairs Office of Research and Development, National Institutes of Health (R15HL141947 and R15HL156214), and funds from the Institutional Research and Improvement account (to KS) and C06RR0306551.

## CONFLICT OF INTEREST

No conflicts of interest, financial or otherwise, are declared by the authors.

## ETHICS STATEMENT

This investigation follows the Guide for the Care and Use of Laboratory Animals published by the US National Institutes of Health (NIH Publication No. 85–23, revised 1996). All experiments were executed following the protocols which were approved by the East Tennessee State University Committee on Animal Care.
